# Gastric EBV‐positive mucocutaneous ulcer: A rare pathological entity

**DOI:** 10.1002/ccr3.2837

**Published:** 2020-04-13

**Authors:** Azza Gabsi, Yosr Zenzri, Ghada Sahraoui, Ihsen Ben Brahim, Mouna Cherif, Yosra Yahyaoui, Nesrine Chraiet, Karima Mrad, Achraf Chedly, Anis Ben Maamer, Amel Mezlini

**Affiliations:** ^1^ Medical Oncology Department Faculty of Medicine of Tunis El Manar University Salah Azaiez Institute Tunis Tunisia; ^2^ Pathology Department Faculty of Medicine of Tunis El Manar University Salah Azaiez Institute Tunis Tunisia; ^3^ Pathology Department Faculty of Medicine of Tunis El Manar University Habib Thameur Hospital Tunis Tunisia; ^4^ Surgery department Faculty of Medicine of Tunis El Manar University Habib Thameur Hospital Tunis Tunisia

**Keywords:** Epstein‐Barr virus, gastrointestinal, mucocutaneous, ulcer

## Abstract

The gastric location of EBVMCU is extremely rare. The pathology examination and immunochemistry are mandatory for the diagnosis. It is essential that physicians be aware of this new entity to accurately diagnose and handle this disease.

## Graphical abstract

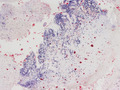

## 1 INTRODUCTION

Epstein‐Barr virus‐positive mucocutaneous ulcer occurs in mucosal sites or skin areas. The gastric location is extremely rare. We report the case of a 62‐year‐old man with gastric EBV‐positive mucocutaneous ulcer. We analyze through this observation the clinical, endoscopic, histological, and therapeutic characteristics of this entity.

Epstein‐Barr virus (EBV)‐positive mucocutaneous ulcers (EBVMCU) were defined for the first time in 2010 and share histological features with other B‐cell proliferative neoplasms.^1^ It has been reported in the setting of iatrogenic immunosuppression and immunosenescence, affecting the oropharyngeal mucosa, skin, and gastrointestinal tract (GIT). We present here an extremely rare case of a EBVMCU of the stomach.

## 2 CASE PRESENTATION

A 62‐year‐old man with a history of heavy smoking (94 pack‐year smoking history) and brain attack was complaining of paroxystic epigastric pain, weight loss, and anorexia since 3 months.

The patient has no medical history suggestive of autoimmune disease, HIV, or other infections. Complete skin examination was performed. There was no cutaneous or nodal involvement. He did not have hepatosplenomegaly or abdominal mass. His laboratory evaluation was negative for HIV, anti‐HBsAg, and HCV antibodies.

**Figure 1:**
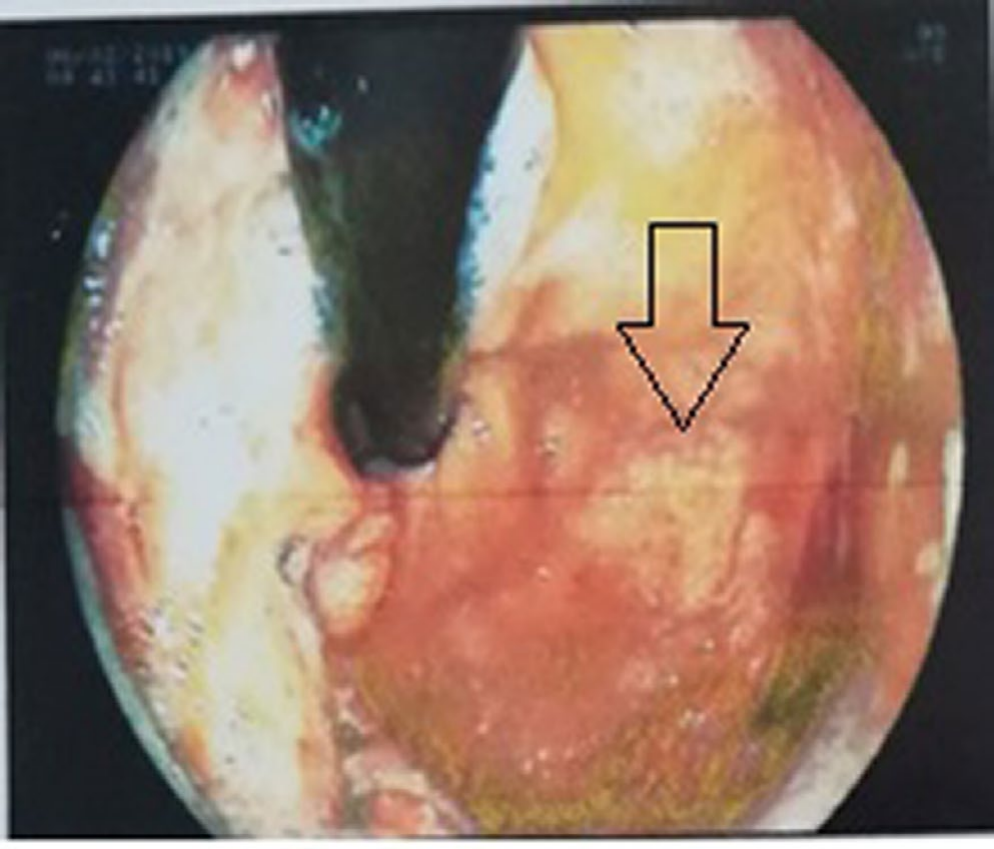
A subcardial ulcerous budding lesion in the greater stomach tuberosity

**Figure 2:**
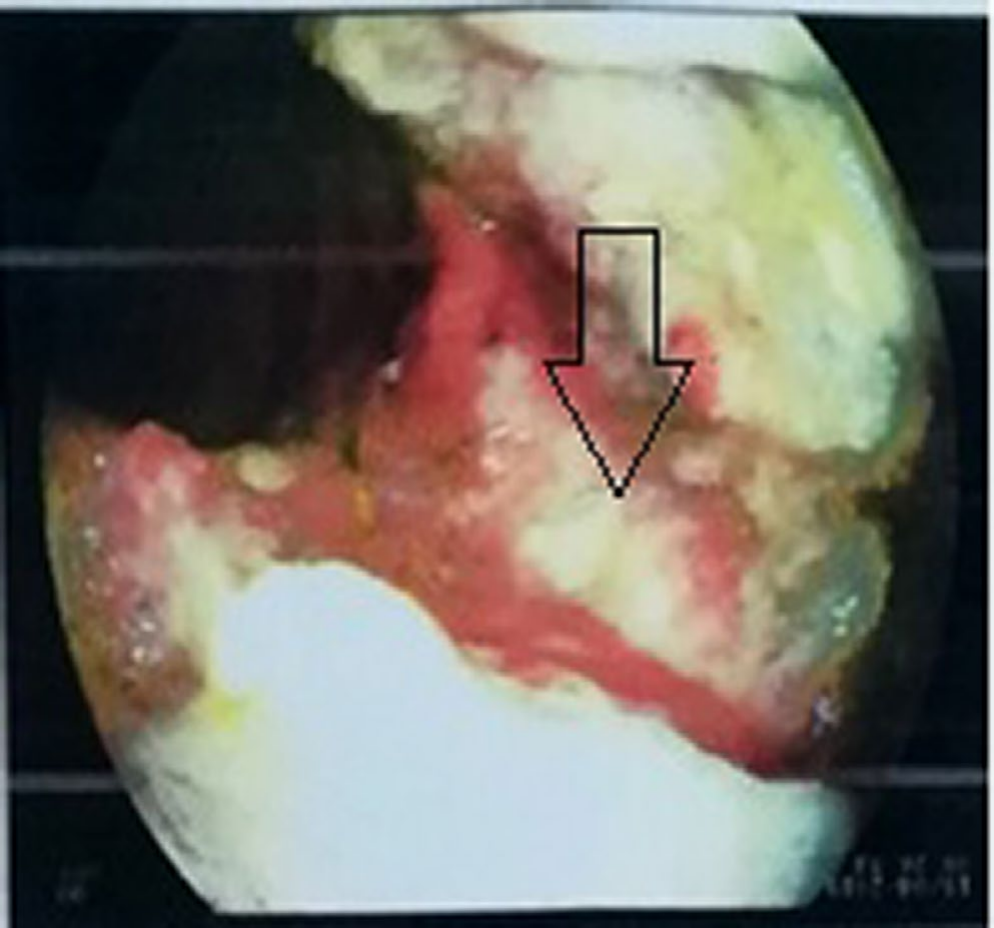
Large ulcerous lesion in the lesser curvature

**Figure 3:**
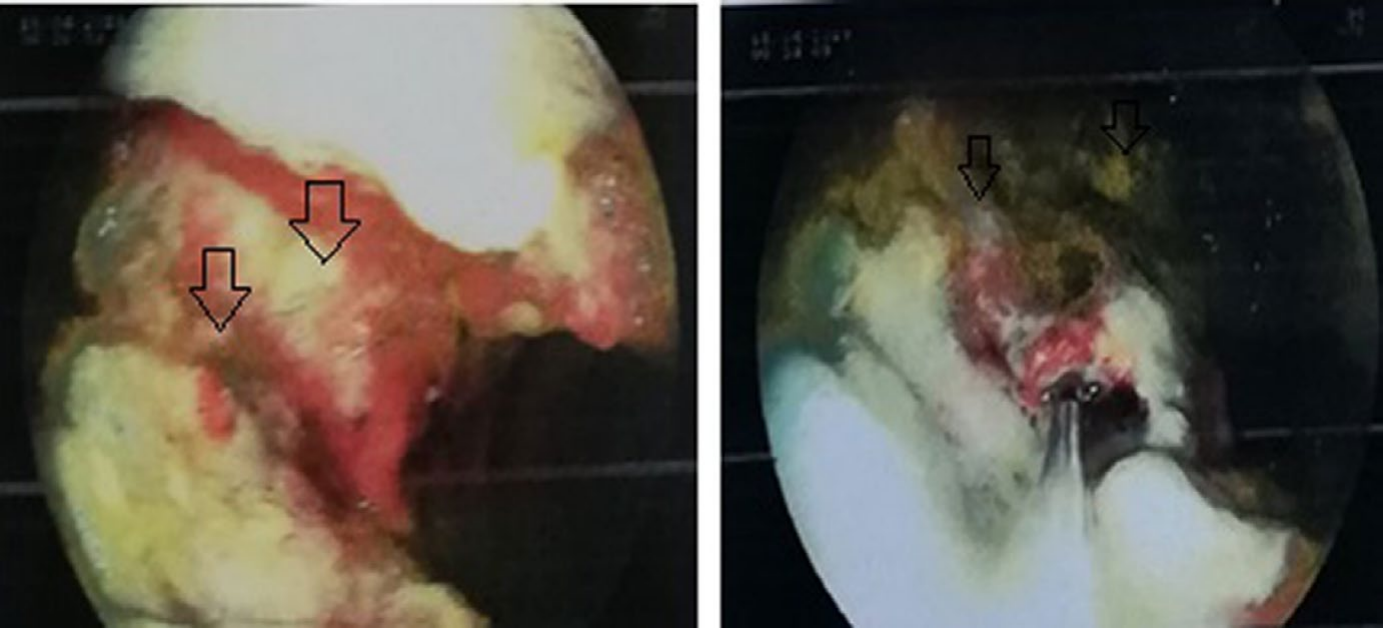
Multiple ulcers were observed in the gastric body and the fundus

**Figure 4:**
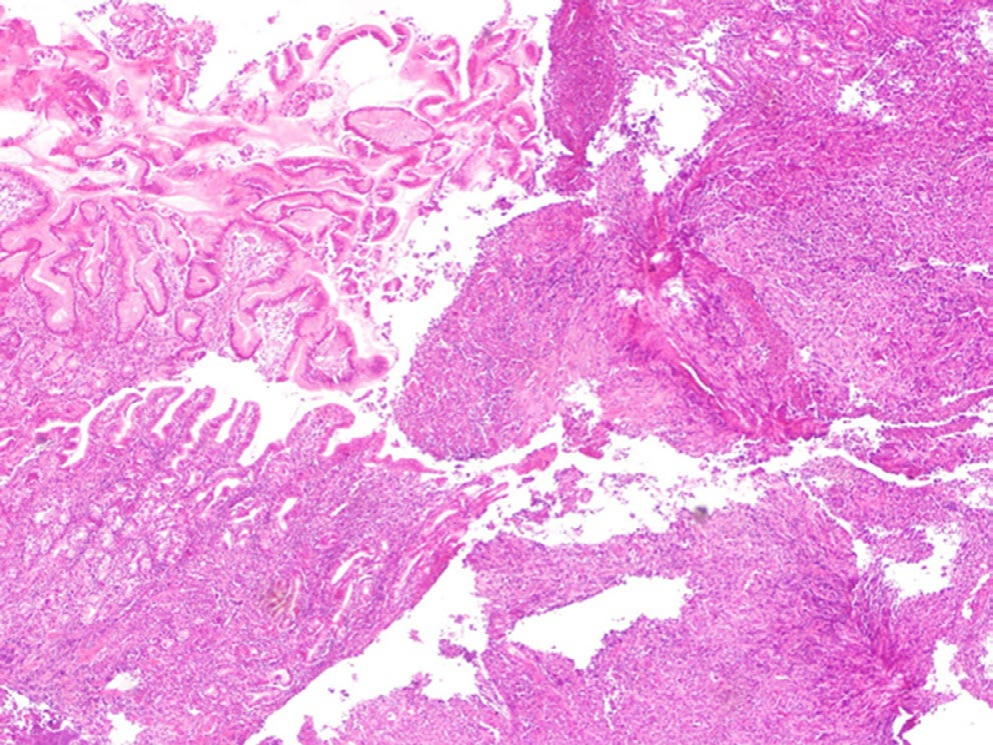
Extensive ulceration with fibrin and leukocyte exudates in the gastric mucosa

**Figure 5:**
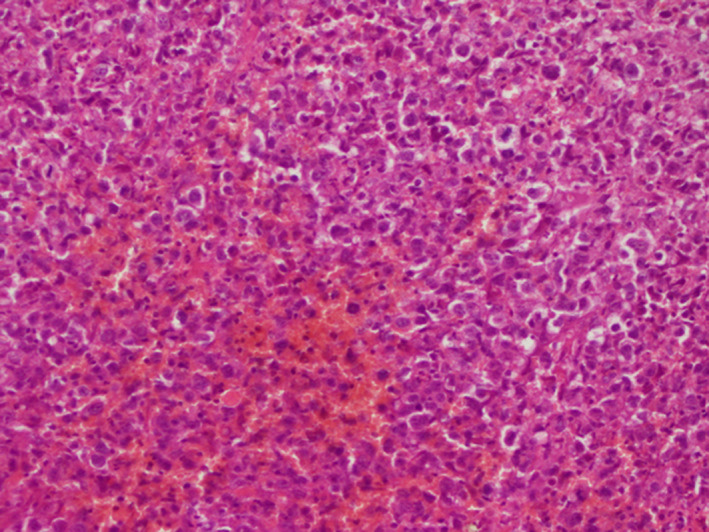
Atypical cells with basophilic cytoplasm resembling Hodgkin Reed‐Sternberg cells associated with lymphocytes, histiocytes, plasma cells, and neutrophils (G ×40)

**Figure 6:**
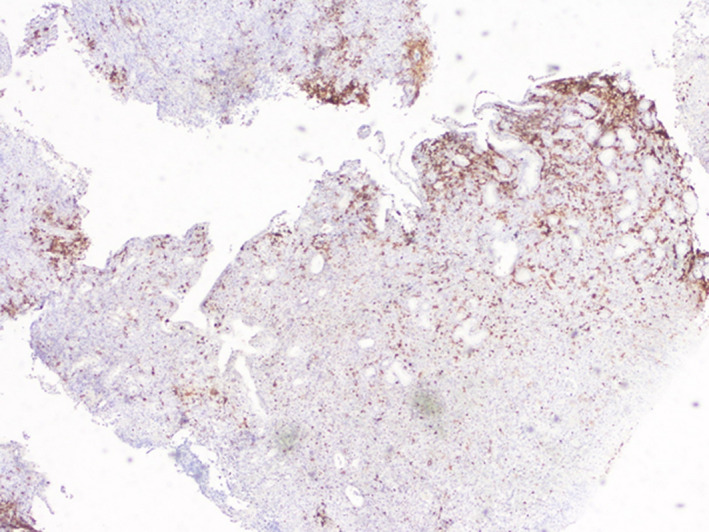
Heterogeneous expression of CD15

**Figure 7:**
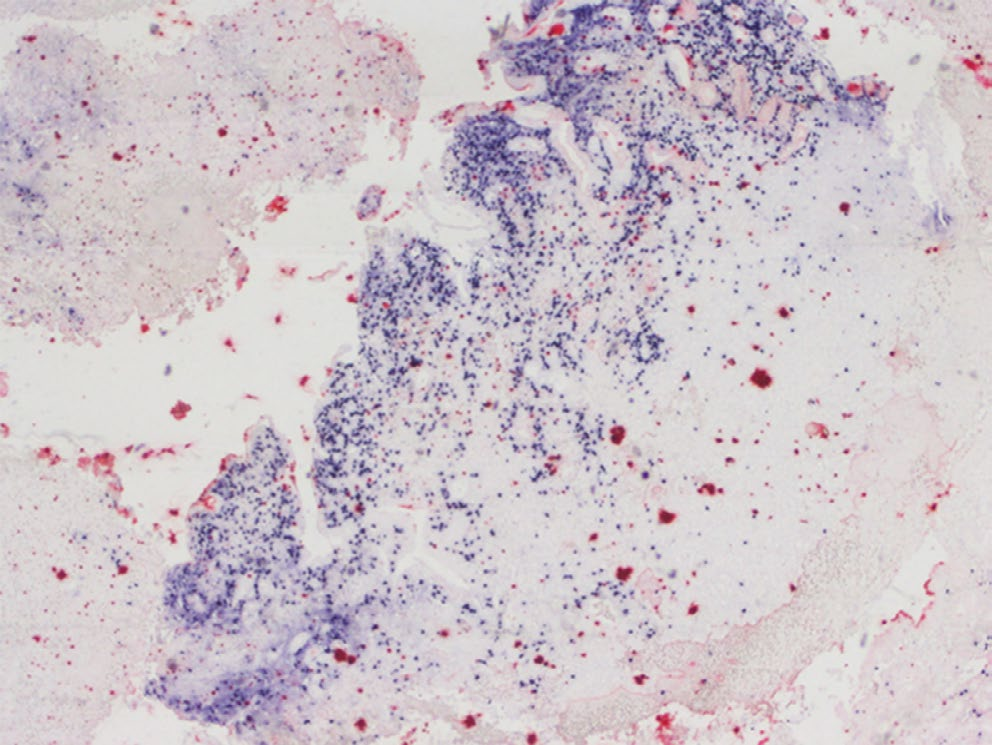
Cells positive to EBV

**Figure 8:**
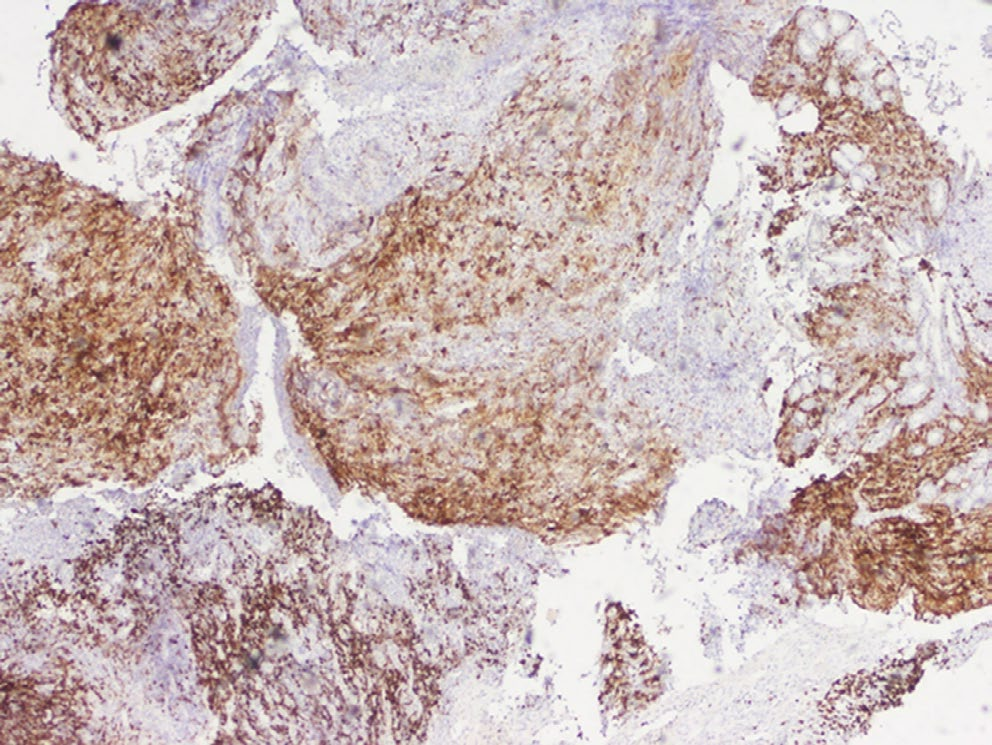
Heterogeneous expression of CD20

An upper gastrointestinal endoscopy was performed. A subcardial large ulcerous budding lesion in the greater stomach tuberosity was observed (Figure 1). Another similar lesion was found in the lesser curvature (Figure 2). Multiple ulcers were observed in the gastric body and the fundus (Figure 3). Histological examination showed an extensive ulceration with fibrin and leukocyte exudates (Figure 4). Atypical cells with basophilic cytoplasm resembling Hodgkin Reed‐Sternberg cells associated with lymphocytes, histiocytes, plasma cells, and neutrophils were observed (Figure 5). Immunohistochemistry was performed using monoclonal antibodies against CD20, CD30, CD15 (Figure 6), EBV, CD3, CD138, and C‐Myc. Immunohistochemical analysis of our patient depicted large cells positive for CD30, CD15, EBV (Figure7), and CD20 (Figure 8) whose expression was heterogeneous.

**Figure 9:**
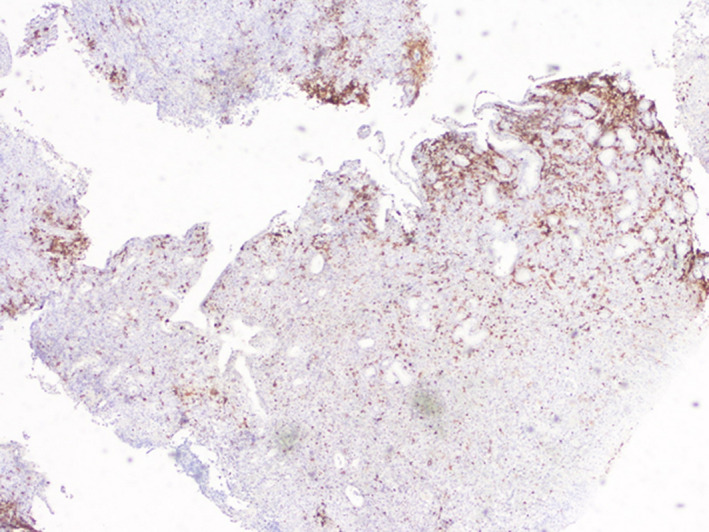
CD3 marks reactive T lymphocytes

CD3 marks reactive T lymphocytes (Figure 9).

Computed tomography (CT) of neck, chest, abdomen, and pelvis revealed diffuse thickening of the gastric wall, especially fundic and antral.

The differential diagnosis of EBVMCU includes diffuse large B‐cell lymphoma (DLBCL) associated with EBV. The patient underwent four cycles of CHOP (doxorubicin, cyclophosphamide, vincristine, and prednisone) without rituximab due to its initial unavailability. CT scan and upper gastrointestinal endoscopy with biopsy showed stable disease. He is undergoing R‐CHOP chemotherapy.

## 3 DISCUSSION

We describe a case of localized EBVMCU in a 62‐year‐old man with atypical and Hodgkin‐like cells. EBVMCU have been recently described and are recognized by lymphoproliferative ulcerated skin or mucosal lesions with absence of hepatosplenomegaly and lymph nodes. This entity is usually associated with primary immunodeficiency, HIV infection, or immunosenescence but also in patients with iatrogenic immunosuppression such as methotrexate or tumor necrosis factors (TNF) inhibitors.^2^ Elderly patients without a history of immunosuppression like our patient can develop EBVMCU.^3^ EBVMCU generally presents with a solitary lesion but can be multifocal in 17% of cases in the literature. It usually occurs in the skin (29%), oral mucosa (52%), or gastrointestinal tract (19%‐40% colon, 30% esophagus, 20% rectum, and 10% terminal ileum).^4^

Since 2010, about 121 cases have been reported in the literature.^5^

No cases of gastric EBVMCU were described in the literature. Our case in the first reported EBV‐positive mucocutaneous ulcer localized in the stomach.

Histological examination usually shows sharply circumscribed ulcer with a polymorphous infiltrate containing a variable number of plasma cells, eosinophils, histiocytes, and large transformed cells resembling Hodgkin Reed‐Sternberg (HRS). Angiovasion and necrosis can be observed.^6^

The HRS‐like cells and large transformed immunoblasts have CD20 expression and lacking expression of CD10 and BCL6. CD20 expression is variable ranging from strong to weak. CD30, EBER, PAX5, MUM1, and OCT2 are positive.^7^ But, there are many differential diagnosis like classic Hodgkin lymphoma (cHL). Immunohistochemical findings can be helpful to confirm the diagnosis. Indeed, abundant CD8‐positive T cells and a large number of EBV‐positive cells as highlighted by CD20, CD30, and EBER are not found in cHL. Moreover, small lymphocytes, immunoblasts, as well as large HRS‐like cells which, unlike in cHL, are CD45positive.^1^ EBV‐positive diffuse large B‐cell lymphoma (DLBCL) is another differential diagnosis, which has a poorer prognosis than EBVMCU. The background consists mainly in lymphocytes, plasma cells, and histiocytes. Cells express B‐cell antigens, CD30, and EBV.^2^

The therapeutic approach in EBVMCU depends on many factors. EBVMCU can be a benign condition that does not require treatment and have some degree of spontaneous regression following cessation or reduction of the immunosuppressive treatment. Rare cases of relapse or progression have been reported.^1^

In some cases, it necessitates a systemic treatment associated with rituximab, radiation therapy, surgery, or combined‐modality therapy.

## 4 CONCLUSION

EBVMCU is a rare newly recognized entity. Clinical history, pathological examination, and immunohistochemistry are necessary to a conclusive diagnosis due to its histopathological similarity with some EBV‐associated malignant lymphoproliferative disorders. Multidisciplinary approach is mandatory.

## CONFLICT OF INTEREST

None declared.

## AUTHOR CONTRIBUTIONS

AG: contributed to guarantor, manuscript review, manuscript editing, manuscript preparation, definition of intellectual content, design, and concepts. YZ: contributed to design, definition of intellectual content, literature search, manuscript preparation, and manuscript editing. GS: contributed to manuscript preparation, definition of intellectual content, manuscript review, and manuscript editing. IBB: contributed to manuscript preparation. NC: contributed to manuscript preparation. MC: contributed to manuscript preparation. KM: contributed to manuscript preparation. AC: contributed to manuscript preparation. YY: contributed to manuscript preparation.ABM: contributed to manuscript preparation. AM: contributed to manuscript review.
